# Supplementary material for: SyNDI: synchronous network data integration framework
Source: BMC Bioinformatics. 2018 Nov 6;19:403. doi: 10.1186/s12859-018-2426-5 (PMC6219086; doi:10.1186/s12859-018-2426-5)
Supplement: Supplementary file 5 — Files of the example presented in Additional file 4. This additional file is a zip package that contains all files used in Additional file 4. Detailed descriptions of each of these files are presented in a README file included in this zip package. (GZ 30411 kb) [file 12859_2018_2426_MOESM5_ESM.gz › SyNDITools/Meme2Fimo/meme_4.9.0_4.tar.gz!/meme_4.9.0/doc/gomo_highlight.html]

gomo\_highlight


# gomo\_highlight

**Usage:**
`gomo_highlight <GO DAG> <GOMO XML>+`

**Description:**

Updates the GOMO XML file(s) to add details about the GO terms, such as their name, hierarchy, relative position in that
hierarchy and which are implied by others in the significant terms for each motif. This allows many improvements to be
made in the human readable html output such as allowing the most specific terms to be highlighted. The same postprocessing
can be achieved by using the --dag option to gomo.

**Input:**

- <GO DAG> - A file containing the heirarchy of the GO terms in the format specified here.
- <GOMO XML> - An xml file output from gomo.

**Output:**

The GOMO XML file(s) are updated.

**Warning messages:**

None

**Bugs:**
None known.
